# Placebo Analgesia Changes Alpha Oscillations Induced by Tonic Muscle Pain: EEG Frequency Analysis Including Data during Pain Evaluation

**DOI:** 10.3389/fncom.2016.00045

**Published:** 2016-05-10

**Authors:** Linling Li, Hui Wang, Xijie Ke, Xiaowu Liu, Yuan Yuan, Deren Zhang, Donglin Xiong, Yunhai Qiu

**Affiliations:** ^1^Research Center for Neural Engineering, Shenzhen Institutes of Advanced Technology, Chinese Academy of SciencesShenzhen, China; ^2^Shenzhen College of Advanced Technology, University of Chinese Academy of SciencesShenzhen, China; ^3^Department of Pain, Shenzhen Sixth People’s Hospital (Nanshan Hospital), Guangdong Medical CollegeShenzhen, China

**Keywords:** placebo, EEG, tonic muscle pain, pain perception, alpha oscillation

## Abstract

Placebo exhibits beneficial effects on pain perception in human experimental studies. Most of these studies demonstrate that placebo significantly decreased neural activities in pain modulatory brain regions and pain-evoked potentials. This study examined placebo analgesia-related effects on spontaneous brain oscillations. We examined placebo effects on four order-fixed 20-min conditions in two sessions: isotonic saline-induced control conditions (with/without placebo) followed by hypertonic saline-induced tonic muscle pain conditions (with/without placebo) in 19 subjects using continuous electroencephalography (EEG) recording. Placebo treatment exerted significant analgesic effects in 14 placebo responders, as subjective intensity of pain perception decreased. Frequency analyses were performed on whole continuous EEG data, data during pain perception rating and data after rating. The results in the first two cases revealed that placebo induced significant increases and a trend toward significant increases in the amplitude of alpha oscillation during tonic muscle pain compared to control conditions in frontal-central regions of the brain, respectively. Placebo-induced decreases in the subjective intensity of pain perception significantly and positively correlated with the increases in the amplitude of alpha oscillations during pain conditions. In conclusion, the modulation effect of placebo treatment was captured when the pain perception evaluating period was included. The strong correlation between the placebo effect on reported pain perception and alpha amplitude suggest that alpha oscillations in frontal-central regions serve as a cortical oscillatory basis of the placebo effect on tonic muscle pain. These results provide important evidence for the investigation of objective indicators of the placebo effect.

## Introduction

Placebo effects on pain perception were characterized using numerous hemodynamic (e.g., functional magnetic resonance imaging (fMRI) and positron emission tomography (PET)) and electrophysiological (e.g., electroencephalography (EEG) and magnetoencephalography (MEG)) in previous studies (Wager et al., 2004, 2006; Lorenz et al., 2005; Zubieta et al., 2005; Scott et al., 2008; Tracey, 2010). Most of these studies demonstrated that placebo analgesia significantly decreased neural activities in pain modulatory brain regions, including thalamus, insula, and anterior cingulate cortex (ACC; Wager and Fields, 2011). Laser-evoked potentials (LEPs) are one of the best tools to assess the function of nociceptive pathways in physiological and clinical settings (Bromm and Treede, 1991; Iannetti et al., 2001), and LEPs were used in previous studies to investigate placebo analgesia (Wager et al., 2006; Watson et al., 2007). These studies demonstrated a clear decrease in P2 amplitude using LEPs (Wager et al., 2006), which suggests that the placebo treatment affected early nociceptive processing (e.g., attention and affect). One recent study reported that placebo analgesia during phasic pain was associated with changes in pain-evoked potentials but not oscillatory activities (Tiemann et al., 2015).

Reports of placebo effects in healthy subjects were primarily based on duration limited phasic pain (Atlas et al., 2009; Benedetti, 2009). Phasic pain provides some important methodological benefits (e.g., safe and easy to apply repeatedly), but it is too short to faithfully simulate clinical pain, which is rarely brief and exhibits an explicit onset of pain perception. Therefore, several studies proposed tonic pain models, which are crucial to model the pain experience in clinical settings (Le Pera et al., 2000; Chang et al., 2001, 2002, 2003, 2004; Huber et al., 2006; Dowman et al., 2008; Nir et al., 2010). One tonic pain model uses pain originating from deep tissue, such as intramuscular infusions of capsaicin or hypertonic saline, which is most frequently encountered in clinical practice pain (Apkarian et al., 2005). The present study used a prolonged muscle infusion of hypertonic saline to generate tonic muscle pain (Stohler and Kowalski, 1999). Hypertonic saline was continuously infused to maintain a relatively stable pain sensation based on real-time feedback of subjective pain intensity (Stohler, 1992).

We collected continuous EEG data during tonic muscle pain to assess the effect of placebo treatment on: (1) the subjective perception of tonic pain; (2) the electrophysiological oscillatory activities; and (3) the correlations between changes in pain perception and oscillatory activities.

## Materials and Methods

### Subjects

The study included 19 subjects (3 females and 16 males, mean age: 23 ± 2 years). All subjects were nonsmokers with no personal history of any neurological or psychiatric disease. None of the subjects had any history of chronic or acute pain up to 4 weeks before and during the study period, and none of the subjects was on any medication. All subjects provided informed consent, and the Human Research Ethics Committee of the Shenzhen Institutes of Advanced Technology, Chinese Academy of Sciences approved the experimental procedures.

### Experimental Design

**Figure 1:**
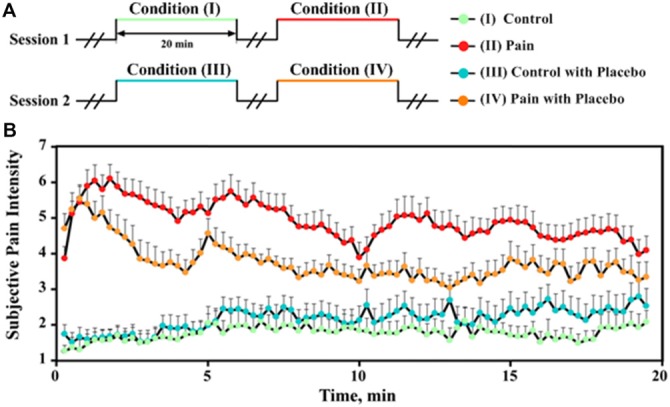
**Experimental design and subjective pain intensity perception. (A)** The experiment consisted of four order-fixed 20-min conditions in two sessions. Session 1: (I) control, (II) pain; Session 2: (III) control with placebo, (IV) pain with placebo. Innocuous and noxious stimulations were respectively applied in control conditions (I and III) and pain conditions (II and IV). **(B)** The subjective pain intensity (mean ± SEM) was collected every 15 s for each condition from all placebo responders (*N* = 14).

The experiment consisted of four order-fixed 20-min conditions in two sessions (Figure 1A): session 1: (I) control, (II) pain; and session 2: (III) control with placebo, (IV) pain with placebo. Subjects were informed that the impending sequential intramuscular injections were possibly painful or non-painful before each session. Experiments were conducted in a silent and separate room, and subjects were comfortably seated in a chair. Subjects were required to rate the intensity of pain perception every 15 s on a computer-controlled visual analog scale (VAS) ranging from 0 to 10 (0: no pain; 10: the most pain intensity imaginable) during all conditions. A moving bar was used to indicate VAS ratings, which were displayed on a monitor in front of the subjects. Subjects indicated the intensity of pain perception by pressing a keyboard key to stop the moving bar with their left hand (the moving bar ascended one score per second). Subjects were asked to arbitrarily choose given scores on the VAS every 15 s until their response was sufficiently accurate to familiarize subjects with the rating paradigm.

We used an automated stimulus delivery system in this study. We used two 24-gauge needles, and each needle was attached to a syringe through a disposable tube. The outline of the masseter muscle was established during clenching. The needles were inserted in bilateral masseter muscles to a depth of approximately 1 cm. Prolonged innocuous stimulation was introduced during control conditions (I and III) via infusions of medication-grade isotonic saline (0.9% NaCl) in the right masseter muscle. During pain conditions (II and IV), prolonged noxious stimulation was introduced by infusing hypertonic saline (5% NaCl) in the left masseter muscle. Automated syringe infusion pumps controlled the infusions. Isotonic saline was infused at a constant speed of 75 μl/min during innocuous stimulation (1500 μl in total). Noxious stimulation included a 0.2-ml bolus infusion over 15 s at the beginning and subsequent continuous infusions at variable speeds (2134 ± 930 μl in total). The speed of infusion was adjusted using a computer-controlled closed-loop system based on the real-time feedback of pain perception to ensure perceived pain intensity maintained at an approximate VAS level of 5 (Zhang et al., 1993; Stohler and Kowalski, 1999). The adaptive controller identified the system dynamic response and proportional-integral-derivative (PID) controller parameters from the subjects’ initial response to the bolus infusion (Zhang et al., 1993). The intramuscular infusion of hypertonic saline produced a deep aching sensation that was similar to chronic muscle pain, and the generated pain sensation disappeared 5–10 min after cessation of the hypertonic saline infusion (Stohler and Kowalski, 1999; Zubieta et al., 2005). Consecutive sessions were separated by at least 10 min.

Subjects were infused with isotonic saline (0.9% NaCl) via an antecubital intravenous port in their right upper limb during all four conditions. However, the subjects were told that the isotonic saline was replaced by a novel medication named “Entacapone” prior to conditions with placebo (III and IV), and they were further given the following clinical trial-type instruction: “we are studying the analgesic effect of a novel medication named “Entacapone”, and it may or may not ease your pain” (Zubieta et al., 2005; Scott et al., 2007). The same infusion profile of noxious stimulation was applied for pain (II) and pain with placebo (IV) for each subject (Scott et al., 2008).

Subjects were instructed to fill out the Chinese version of the Positive and Negative Affective Scale (PANAS; Watson et al., 1988) and McGill Pain Questionnaire (SF-MPQ; Melzack, 1987) after each condition (I–IV) to provide details of their subjective perceptions of pain. The Chinese version of these questionnaires exhibits acceptable reliability and validity (Huang et al., 2003; Li et al., 2013).

### Behavioral Data Analysis

The average rating of pain intensity across all rating points (once every 15 s) was calculated for each subject during each condition. Subjects who reported an increase in the average rating of the intensity of pain perception to noxious stimulation after the placebo treatment (II vs. IV) were classified as nocebo responders, and the other subjects were classified as placebo responders (Scott et al., 2008). Previous studies reported that placebo and nocebo effects were associated with opposite responses of dopamine and endogenous opioid neurotransmission in a distributed network of cortical and subcortical regions (Scott et al., 2008). Therefore, psychophysical and electrophysiological data from nocebo responders were excluded from subsequent analyses.

Psychophysical data analyses were performed as follows. The ratings of pain perception, positive affect ratings (PANAS-P) and negative affect ratings (PANAS-N) were compared across all four conditions using a two-way repeated-measures analysis of variance (RM ANOVA), with “pain” (two levels: control vs. pain) and “placebo” (two levels: without vs. with placebo) as factors. *Post hoc* tests were performed when the interaction effect was significant. Not all subjects finished the SF-MPQ questionnaire after control conditions (I and III), so the total MPQ sensory (MPQ-S) and affective (MPQ-A) scores of only pain conditions (II and IV) were calculated for each subject. The scores were compared between two pain conditions using a two-tailed paired sample *t*-test.

### EEG Recording and Data Analysis

Continuous EEG data were recorded using a Neuroscan^®^ Scan 4.2 (Neuroscan, Charlotte, NC, USA) amplifier and 128 Ag/AgCl electrodes mounted on an elastic cap (Quickap^®^, Neuromedical supplies, Charlotte, NC, USA) according to the extended international 10–20 system (Aslaksen et al., 2007). The reference channel was located at the vertex, and all channel impedances were kept lower than 10 kΩ. Extracranial activity was continuously recorded with a 0.05 Hz and 100 Hz band-pass filter and was digitized at a sampling rate of 1000 Hz. A notch filter was set to 50 Hz to reduce electrical interference. Electro-oculographic (EOG) signals were simultaneously recorded from four surface electrodes (one pair over the upper and lower eyelids; the other pair placed 1 cm lateral to the outer corner of the left and right orbits) to monitor ocular movements and eye blinks. Subjects were instructed to relax and keep their eyes open during each condition.

#### Preprocessing

EEG data were analyzed using Matlab (The Mathwork, Natick, MA, USA) and EEGLAB^1^, which is an open source toolbox running under the Matlab environment. Continuous EEG data for each condition were down-sampled to 500 Hz and band-pass filtered between 1 and 100 Hz. Continuous EEG data contaminated by eye-blinks and movements were corrected using an independent component analysis (ICA) algorithm (Makeig et al., 1997; Jung et al., 2001; Delorme and Makeig, 2004). The de-noised EEG data were re-referenced to a common average reference. EEG data collected during a short period of 30 s at the beginning and end of each condition were discarded to exclude possible brain responses related to the sudden change in stimulation.

#### EEG Spectral Analysis

Nineteen minutes of continuous EEG data from each subject and condition were transformed to the frequency domain using a discrete Fourier transform to yield amplitude spectra (in μV) ranging from 1 to 100 Hz. The amplitudes of EEG oscillations in the delta (0−4 Hz), theta (4−8 Hz), alpha (8−12 Hz), beta (12−30 Hz), and gamma (30−100 Hz) bands were calculated for each condition and electrode, and the first group of amplitude spectra was obtained.

Previous studies generally used verbal pain perception ratings. EEG data during ratings were excluded because of the possible confounding factor of speaking. Subjects in this experiment indicated the intensity of pain perception by pressing a keyboard key to stop a moving bar with their left hand. This pain rating procedure required a longer time than verbal pain rating because the moving bar ascended one score per second. We investigated whether the inclusion of the EEG data during pain perception was important for the extraction of placebo-related modulation effects. Therefore, additional separate analyses were performed with the EEG data during pain perception rating and EEG data after rating. Pain perception ratings were repeated once in every 15 s. Subjects pressed a button when the moving VAS bar indicated their pain intensity. Therefore, we partitioned the EEG data based on the time point when the pain intensity rating was completed. The original preprocessed continuous EEG data were segmented into EEG epochs of 1 s, and the segmented EEG epochs were transformed to the frequency domain for each subject and each condition to facilitate the partition. The obtained single-epoch amplitude spectra according to time period during rating were averaged for each electrode and condition to provide another group of amplitude spectra. The numbers of segments during ratings were 2.60 ± 1.17, 5.39 ± 1.19, 2.25 ± 1.15 and 4.19 ± 0.70 in conditions I, II, III, and IV, respectively. The obtained single-epoch amplitude spectra according to time periods after VAS rating were also extracted to provide a third group of amplitude spectra. The numbers of segments after ratings were 12.40 ± 1.17, 9.61 ± 1.19, 12.75 ± 1.15 and 10.81 ± 0.70 in conditions I, II, III, and IV, respectively.

All three groups of amplitude spectra were compared across all four conditions using point-by-point two-way RM ANOVA with “pain” (two levels: control vs. pain) and “placebo” (two levels: without vs. with placebo) as factors. Considering the two-by-two experimental design, significant interaction effect indicated the placebo effect. A permutation test with 5000 iterations was used to construct the null distribution of the max F-statistic across electrodes to control for multiple comparisons. We identified the F-statistic that corresponded to the 5% most extreme parts of the maximal F distribution. We thresholded our original statistical maps at that 5% level from the maximal F distribution (Maris and Oostenveld, 2007). Compared with this F-statistic, higher *F* value represented significant result after correction. Besides, we calculated the corrected *P* value of our observed *F* value by counting the proportion of the permutation distribution as or more extreme than F. Results of main effects and *post hoc* tests were presented when the interaction effect was significant.

#### Correlation Analysis

The correlation coefficients and significance of placebo responders were calculated between changes in the amplitude of alpha oscillation measured at frontal-central electrode FCz after placebo treatment (IV–II) and changes in: (1) subjective intensity of pain perception to noxious stimulation; and (2) psychophysical scores (i.e., PANAS and MPQ scores; II–IV). Besides, in order to keep consistent with the two-by-two experimental design, correlation analysis was also performed with changes which were calculated according to the interaction effect ((IV–II)–(III–I)) for alpha amplitude; ((II–IV)–(I–III)) for subjective intensity).

## Results

### Psychophysical Results

The subjective intensity of pain perception to noxious stimulation increased after the placebo treatment (IV vs. II) in five subjects (nocebo responders) and decreased in the remaining 14 subjects (placebo responders). The intensity of pain perception to noxious stimulation for placebo responders revealed an overall declining tendency with increased stimulus duration (II and IV; Figure 1B), which may be due to the limitation of the maximum speed of hypertonic saline infusion. In contrast, the intensity of pain perception to innocuous stimulation was approximately a VAS level of 2 and increased slightly with increased stimulus duration (I and III; Figure 1B), which may be caused by the needle effect (Veerasarn and Stohler, 1992).

**Figure 2:**
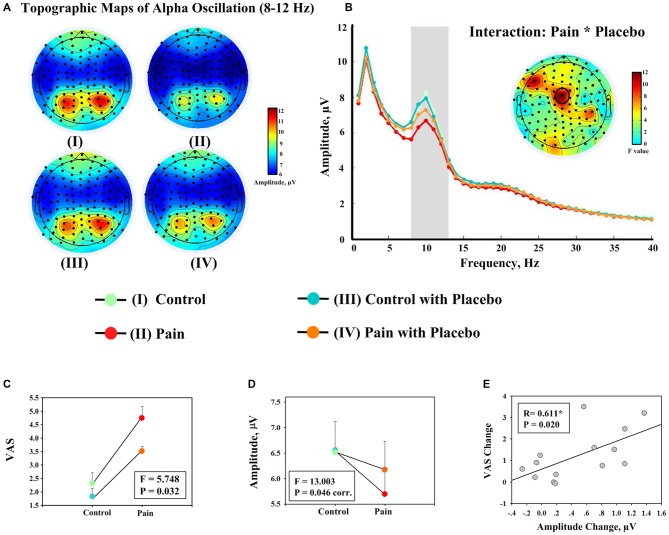
**Evidence showing the effect of placebo treatment from behavioral and EEG data. (A)** Group level scalp topographies of alpha oscillations (8–12 Hz) of different experimental conditions. **(B)** Group level spectra (measured at FCz) of different experimental conditions. Scalp topography showing the significant interaction between the factors “pain” and “placebo” on the amplitudes of alpha oscillations at FCz is displayed in the insert. **(C)** Significant interaction effect between the factors “pain” and “placebo” was observed on the average ratings of pain intensity across all rating points (once every 15 s; left). **(D)** The amplitudes of alpha oscillation (measured at FCz). Each dot represents the mean value from one condition, and error bars represent, for each condition, ± SEM across subjects (F: *F* value of the interaction effect between the factors “pain” and “placebo”; corr.: corrected for multiple comparisons). **(E)** Significant correlation was observed between decrease in pain intensity during noxious stimulation after placebo treatment (II–IV) and the increase in the amplitude of alpha oscillation measured at FCz (IV–II). Each dot represents a value from each subject, and black line represents the best linear fit.

Table 1 summarizes the average ratings of subjective pain intensity, PANAS scores, and MPQ scores for placebo responders. The intensity of pain perception was significantly modulated by the factors “placebo” (*F*_(1,13)_ = 25.889, *P* = 0.000) and “pain” (*F*_(1,13)_ = 105.663, *P* = 0.0000) and the interaction between two factors (*F*_(1,13)_ = 5.748, *P* = 0.032; Figure 2C). The decrease in pain intensity to noxious stimulation was significant after placebo treatment (II vs. IV; *P* = 0.000), but only marginally significant to innocuous stimulation (I vs. III; *P* = 0.058). The PANAS-P scores were not significantly modulated by the factor “pain” (*F*_(1,13)_ = 1.050, *P* = 0.324) or “placebo” (*F*_(1,13)_ = 2.444, *P* = 0.142), or the interaction between the two factors (*F*_(1,13)_ = 0.918, *P* = 0.356). The PANAS-N scores were significantly modulated by the factor “placebo” (*F*_(1,13)_ = 8.050, *P* = 0.014) but not the factor “pain” (*F*_(1,13)_ = 1.518, *P* = 0.240) or the interaction between the two factors (*F*_(1,13)_ = 1.194, *P* = 0.294). MPQ-S scores decreased significantly in condition IV compared to condition II (*t*_(13)_ = 2.230, *P* = 0.044). In contrast, the MPQ-A scores were not significantly different between conditions II and IV (*t*_(13)_ = 1.906, *P* = 0.079).

**Table 1:** **Psychophysical responses of placebo responders**.

	Pain Intensity	PANAS-P	PANAS-N	MPQ-S	MPQ-A
**Condition I**	2.16 ± 1.17	23.14 ± 6.13	14.64 ± 3.88	−	−
**Condition II**	4.93 ± 1.21	21.36 ± 6.49	16.50 ± 5.49	9.93 ± 6.06	6.21 ± 3.95
**Condition III**	1.79 ± 1.11	20.64 ± 6.38	13.43 ± 3.16	−	−
**Condition IV**	3.75 ± 0.71	20.50 ± 6.47	13.36 ± 2.95	3.36 ± 2.27	2.07 ± 1.64

*Mean ± 1 SD of psychophysical measures during conditions in the absence and presence of placebo in 14 placebo responders. Pain intensity refers to the average ratings of momentary pain acquired every 15 s*.

### Electrophysiological Results

Frequency analyses of the 19-min continuous EEG data revealed that the group level scalp topographies of alpha oscillations were maximal at bilateral posterior parietal and occipital regions in all four conditions (Figure 2A). Point-by-point two-way RM ANOVA revealed that electrode FCz exhibited a significant interaction effect on the amplitudes of alpha oscillations after correction for multiple comparisons (Figure 2B). The amplitudes of alpha oscillations at FCz were 6.56 ± 2.19 μV, 5.70 ± 1.80 μV, 6.52 ± 2.23 μV, and 6.18 ± 2.04 μV in conditions I, II, III, and IV respectively. The amplitudes of alpha oscillations at FCz were significantly modulated by the factor “pain” (*F*_(1,13)_ = 13.886, *P* = 0.040, corr.) and the interaction between the two factors (*F*_(1,13)_ = 13.003, *P* = 0.046, corr.; Figure 2D), but not by the factor “placebo” (*F*_(1,13)_ = 1.483, *P* = 0.864, corr). *Post hoc* tests revealed that the amplitudes of alpha oscillations were significantly larger in condition IV than condition II (*P* = 0.005), but no significant difference was observed between the amplitudes of alpha oscillations in conditions I and III (*P* = 0.846).

**Figure 3:**
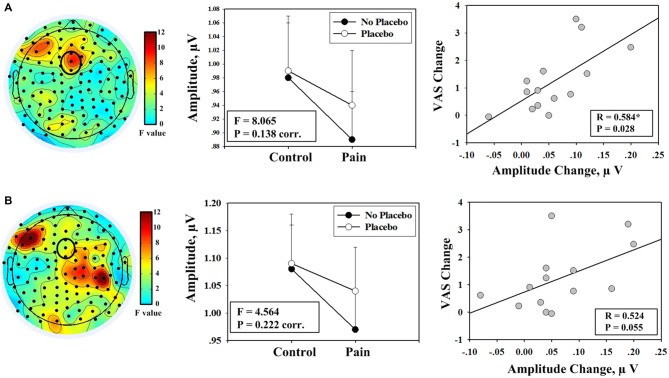
**Evidence showing the effect of placebo treatment from the partitions of EEG data during pain perception rating and after rating. (A)** A trend toward significant interaction effect was identified from frequency analyses including EEG data during the rating period (left panel); the mean values of the amplitudes of alpha oscillation (measured at FCz) from each condition are shown (F: *F* value of the interaction effect between the factors “pain” and “placebo”; corr.: corrected for multiple comparisons; middle panel); a significant correlation was observed between decrease in pain intensity during noxious stimulation after placebo treatment (II–IV) and the increase in the amplitude of alpha oscillation measured at FCz (IV–II; right panel). **(B)** No significant interaction effect was identified from frequency analyses that included EEG data of the time periods after rating (left panel); the corresponding results of mean amplitude values are shown (middle panel); marginally significant correlation was observed (right panel).

Frequency analyses results of EEG data during pain perception ratings revealed that electrode FCz exhibited a trend toward significant interaction effect between the factors “pain” and “placebo” on the amplitudes of alpha oscillation (*F*_(1,13)_ = 8.065, *P* = 0.014, uncorr., *P* = 0.138, corr.; left and middle panels of Figure 3A). *Post hoc* tests revealed that the amplitudes of alpha oscillations were significantly larger in condition IV than condition II (*P* = 0.004), but no significant difference was observed between conditions I and III (*P* = 0.638). Analysis results of EEG data after VAS ratings revealed that the interaction effect was not significant (*F*_(1,13)_ = 4.564, *P* = 0.222, corr.; left and middle panels of Figure 3B).

### Correlation between Psychophysical and Electrophysiological Data

First, the correlation analysis was performed with changes between two pain conditions (II vs. IV). Significant positive correlation was observed between increases in the amplitudes of alpha oscillations measured at FCz after placebo treatment and decreases in: (1) subjective intensity of pain perception (*R* = 0.611, *P* = 0.020; Figure 2E); (2) MPQ-S scores (*R* = 0.641, *P* = 0.014); and (3) MPQ-A scores (*R* = 0.594, *P* = 0.025) when EEG data of the entire 19-min continuous EEG data were included. The correlation between alpha oscillation increases and pain perception decreases was also significant when only EEG data during pain perception rating were included (*R* = 0.584, *P* = 0.028). The correlation was marginally significant when only EEG data after pain perception rating were included (*R* = 0.524, *P* = 0.055). Secondly, no significant correlation was observed when the correlation analysis was performed with the interaction terms (*P* > 0.05).

## Discussion

The present study described an active placebo effect on electrophysiological alpha oscillations during 20 min of tonic muscle pain. We observed placebo effects on the subjective intensity of pain perception to noxious stimulation. Placebo induced significant increases or a trend toward significant increases in the amplitude of alpha oscillation during tonic muscle pain in frontal-central regions when EEG data during pain perception ratings were not excluded. The decreases in the subjective intensity of pain perception to noxious stimulation after placebo treatment and the increases in the amplitude of alpha oscillation were significantly correlated. These findings suggest that placebo modulation in cognitive appraisal/experience of tonic muscle pain were effectively indexed by electrophysiological alpha oscillations, which served as additional evidence for the expectancy-based placebo mechanism (Wager et al., 2004; Zubieta et al., 2005; Scott et al., 2007; Atlas and Wager, 2012).

Numerous neuroimaging studies, including fMRI and PET studies of healthy subjects and clinical patients, revealed several cortical and subcortical regions that were mediated by placebo treatment (Meissner et al., 2011). The placebo analgesia also suppressed pain-induced responses in thalamus, insula, and ACC (Wager et al., 2004; Bingel et al., 2006; Kong et al., 2006; Price et al., 2007; Eippert et al., 2009). Assessments of the placebo effect to LEPs revealed a significant decrease in P2 amplitude, which was partially explained by the reduction in reported pain perception (Wager et al., 2006). The P2 in LEPs is highly likely generated from the ACC (Garcia-Larrea et al., 2003), and the decrease in P2 amplitude is consistent with the suppression of pain-induced responses in the ACC, which provides solid evidence that placebo analgesia is likely achieved via modulation of the emotional and cognitive components of pain (primarily coded by the ACC; Wiech et al., 2008; Tracey, 2010).

The placebo modulation effect that we observed supports the existence of a placebo effect on brain oscillation. The placebo treatment-induced changes in alpha oscillatory activities were maximal at frontal-central electrodes, which suggests the contribution of ACC to the generation of placebo-induced changes in alpha oscillations and confirms the modulation of placebo on the affective and cognitive components of pain that were observed to previous fMRI and PET studies (Wiech et al., 2008; Zubieta and Stohler, 2009; Tracey, 2010). Notably, the suppression of alpha amplitudes may reflect cortical activation or disinhibition of the corresponding neural networks (Pfurtscheller et al., 1996; Pfurtscheller and Lopes da Silva, 1999; Hu et al., 2013). For example, increased cellular excitability in thalamo-cortical systems was reflected by a decrease in alpha amplitude in EEG (Steriade and Llinás, 1988). Thus, the significant increase of alpha amplitude at frontal-central regions after placebo treatment may indicate an inhibition of cortical areas (including ACC) that are involved in pain processing (e.g., cognitive appraisal of tonic pain). However, we cannot make any firm conclusions about the contribution of ACC to the generation of placebo-induced changes in alpha oscillations without source analyses. We also cannot exclude the possible contribution of other neural sources (e.g., operculo-insular cortex) despite the performance of an EEG source analysis because of the limited spatial resolution of the EEG technique and the inverse problem in EEG source analysis (Michel et al., 2004). Hopefully, these issues may be effectively solved using the simultaneous EEG-fMRI technique, which was effectively used to extract fMRI activations that were significantly modulated by the alpha amplitude in EEG (Feige et al., 2005).

Only two published studies reported placebo treatment effects on brain alpha oscillatory activity. One study related alpha activity to placebo analgesia and reported a placebo-associated increase in alpha oscillations (Huneke et al., 2013). However, this study recorded alpha activity during resting states after placebo induction (Huneke et al., 2013). Another study reported that phasic pain-induced alpha responses were not sensitive to placebo manipulation using changes in stimulus intensity (Tiemann et al., 2015). This study did not include EEG data during pain perception (Tiemann et al., 2015). The placebo effect was derived from the cognitive and affective processing of pain perception, which may be more promising during the rating period. The alpha suppression in response to tonic pain primarily reflects high-level cognitive processing, and attention modulation may significantly affect it (Peng et al., 2014). Placebo treatment-related modulation effects of alpha oscillations may be better captured when subjects are asked to focus on their pain perception and report their pain intensity. Consequently, we observed significant modulation effects of placebo treatment and a positive correlation between placebo-induced pain decrease and increase in alpha amplitude when the pain perception evaluating period was included.

This study generated tonic muscle pain via an intramuscular infusion of hypertonic saline to produce a deep aching that was similar to the muscle pain experienced in clinical situations (Stohler and Kowalski, 1999). Our understanding of the neural mechanisms of pain were primarily based on the brain activation of phasic cutaneous pain, which involves fewer methodological challenges (e.g., easier to present several times to achieve a high signal-to-noise ratio of the brain responses) compared to tonic pain (Apkarian et al., 2011). However, chronic pain is normally prolonged and originates from deep tissue (e.g., muscle and viscera) in clinical practice (Apkarian et al., 2005; Schreckenberger et al., 2005). Therefore, the tonic muscle pain achieved by intramuscular infusion of hypertonic saline was used in the present study. The automated stimulus delivery system produced a prolonged, relatively stable muscle pain and achieved a better simulation of the pain experience in clinical settings, which may be important to establish the connection between placebo analgesic studies conducted in experimental settings (healthy subjects) and clinical practice (chronic pain patients).

There are several limitations to this study. First, this study consisted of fixed-order sessions (session 1: conditions I and II, session 2: conditions III and IV). Session 1 was always performed before session 2 because the individual infusion profiles used in condition IV should be identical to condition II. We cannot exclude the confounding factors of mental fatigue-induced alpha oscillation changes in this fixed-order and longer-lasting experiment. Experiments with prolonged stimulation are difficult to control as well as experiments using phasic stimulation. Mental fatigue and its influence on the measures of brain oscillation should be carefully considered. Spectral measures of brain oscillations were investigated to reflect changes in mental state in longer-lasting experiments. Several EEG measures were proposed to be valid and reliable indicators of mental fatigue, including a characterized shift of EEG power towards lower-frequency bands (delta, theta and alpha) and decrease in higher-frequency bands (Lal and Craig, 2002; Wascher et al., 2014). The amount of alpha suppression declined with time on task (Wascher et al., 2014). An increase in alpha power may reflect the increased effort and the difficulty of the subjects to maintain a state of alert wakefulness (Wascher et al., 2014). The significant correlation between the differences in pain perception and alpha amplitude was observed when the differences were calculated between two pain conditions II and IV. But no significant correlation could be observed when the differences were calculated according to the interaction effect. Small sample size and fixed-order design might be some of those factors that contributed to this problem. Therefore, the correlation between the effect size of placebo analgesia in pain perception and alpha amplitude require further investigation using a randomized design and within-subject correlation analysis may offer more solid evidence. Second, the saline infusions in the control conditions and the pain conditions occurred on different sides. Therefore, we could only focus on the results of central electrodes in this study. The acquisitions of EEG data involve up to a few hundred electrodes positioned on the scalp, which together with volume conduction through the head results in a poor spatial resolution (Michel et al., 2004). The spreading effect from the lateral electrodes should be taken into consideration when interpreting the observed effects at the central electrodes. Third, the number of segments was different for different conditions when performing additional analyses with EEG data during and after ratings. This difference may be a confounding factor for comparisons of the amplitude spectrum among four conditions. Fourth, we only performed multiple comparisons correction for the number of electrodes (Schulz et al., 2015), but the correction for point-by-point analysis should account for the number of electrodes and the number of frequency bands (Peng et al., 2014). Previous studies reported an association of placebo and nocebo effects with opposite responses of dopamine and endogenous opioid neurotransmission in a distributed network of cortical and subcortical regions (Scott et al., 2008), and possible electrophysiological responses that are oppositely involved in placebo and nocebo effects should be assessed in the future.

## Author Contributions

LL, HW, XK and YQ designed the study. LL, HW, XK, XL and YY collected the data. LL analyzed the data. LL, DZ, DX and YQ discussed the results and wrote the article.

## Conflict of Interest Statement

The authors declare that the research was conducted in the absence of any commercial or financial relationships that could be construed as a potential conflict of interest.

## Footnotes

^1^http://sccn.ucsd.edu/eeglab
