# Corrigendum: Placebo Analgesia Changes Alpha Oscillations Induced by Tonic Muscle Pain: EEG Frequency Analysis Including Data during Pain Evaluation

**DOI:** 10.3389/fncom.2016.00125

**Published:** 2016-12-14

**Authors:** Linling Li, Hui Wang, Xijie Ke, Xiaowu Liu, Yuan Yuan, Deren Zhang, Donglin Xiong, Yunhai Qiu

**Affiliations:** ^1^Research Center for Neural Engineering, Shenzhen Institutes of Advanced Technology, Chinese Academy of SciencesShenzhen, China; ^2^Shenzhen College of Advanced Technology, University of Chinese Academy of SciencesShenzhen, China; ^3^Department of Pain, Shenzhen Sixth People's Hospital (Nanshan Hospital), Guangdong Medical CollegeShenzhen, China

**Keywords:** placebo, EEG, tonic muscle pain, pain perception, alpha oscillation

Reason for Corrigendum:

In the original article, we have noticed that the affiliation is listed wrongly for Linling Li and Hui Wang as only 1 (Research Center for Neural Engineering, Shenzhen Institutes of Advanced Technology, Chinese Academy of Sciences, Shenzhen, China). The correct affiliation should be Linling Li^1, 2^ and Hui Wang^1, 2^ (1-Research Center for Neural Engineering, Shenzhen Institutes of Advanced Technology, Chinese Academy of Sciences, Shenzhen, China; 2-Shenzhen College of Advanced Technology, University of Chinese Academy of Sciences, Shenzhen, China), Xijie Ke^3^, Deren Zhang^3^ and Donglin Xiong^3^ (3-Department of Pain, Shenzhen Sixth People's Hospital (Nanshan Hospital), Guangdong Medical College, Shenzhen, China). The authors apologize for this oversight. This error does not change the scientific conclusions of the article in any way.

The original article has been updated.

## Conflict of interest statement

The authors declare that the research was conducted in the absence of any commercial or financial relationships that could be construed as a potential conflict of interest. The handling Editor declared a shared affiliation, though no other collaboration, with several of the authors LL and HW and states that the process nevertheless met the standards of a fair and objective review.

